# Overexpressed MicroRNA-182 Promotes Proliferation and Invasion in Prostate Cancer PC-3 Cells by Down-Regulating N-myc Downstream Regulated Gene 1 (NDRG1)

**DOI:** 10.1371/journal.pone.0068982

**Published:** 2013-07-16

**Authors:** Ranlu Liu, Jing Li, Zhigang Teng, Zhihong Zhang, Yong Xu

**Affiliations:** 1 Tianjin Institute of Urology & Department of Urology, Second Hospital, Tianjin Medical University, Tianjin, China; 2 Department of Urology, affiliated cancer hospital of Zhengzhou University, Henan Cancer Hospital, Zhengzhou, China; 3 Department of Urology, Kaifeng People’s Hospital, Kaifeng, China; Southern Illinois University School of Medicine, United States of America

## Abstract

MicroRNAs, non-coding 20–22 nucleotide single-stranded RNAs, result in translational repression or degradation and gene silencing of their target genes, and significantly contribute to the regulation of gene expression. In the current study, we report that miR-182 expression was significantly upregulated in prostate cancer tissues and four cell lines, compared to benign prostatic hyperplasia tissues and normal prostatic epithelial (RWPE-1) cells. Ectopic overexpression of miR-182 significantly promotes the proliferation, increases the invasion, promotes the G1/S cell cycle transition and reduces early apotosis of PC-3 cells, while suppression of miR-182 decreased the proliferation and invasion, inhibits the G1/S cell cycle transition and increase early apotosis of PC-3 cells. Additionally, we demonstrated that miR-182 could downregulate expression of NDRG1 by directly targeting the NDRG1 3′-untranslated region. In conclusion, our results suggest that miR-182 plays an important role in the proliferation of human prostate cancer cells by directly suppressing the tumor supressor gene NDRG1. We uncovered a new epigenetic regulation of NDRG1.

## Introduction

Prostate cancer (PCa) continues to be one of the biggest health problems for the aging male, with an estimated 238,590 new cases and 29,720 cancer related deaths expected in 2013 in the United States alone [1]. Though Chinese men belong to low risk group of having PCa, the incidence and mortality rate have increased significantly due to population aging, changes in life styles and other causes. PCa has become one of the most common malignancies in men worldwide, with strongly varying rates of tumor progression and responses to treatment. If the tumor is confined to the prostate, patients can be treated by surgical removal of the tumor or by radiation, with high efficacy. By contrast, therapy for unconfined tumors still represents a major problem. Standard treatment for these patients is antiandrogens to achieve total androgen blockade [2]. Unfortunately, that tumors progress and thus circumvent the treatment is a very frequent event and there are no effective treatments for castration resistant PCa (CRPC) patients, though urologist are trying to use chemotherapeutics. Although both genetic and environmental factors are considered to be major factors, the molecular mechanisms of PCa development and progression remain largely unknown.Therefore, better understanding the pathogenesis of PCa and exploring novel intervention targets for PCa are urgently demanding tasks.

MicroRNAs (miRNAs) are a group of small (approximately 20–22 nucleotides) endogenous noncoding RNAs. Mature miRNAs negatively regulate their target genes through imperfect complementary sequence pairing to the 3′ untranslated region (UTR) of target genes resulting in either mRNA degradation or translational repression. Numerous studies have demonstrated that aberrant expression of miRNAs is closely associated with proliferation, invasion, metastasis and the prognosis of various cancers, including PCa, breast cancer, glioma and lung cancer [3]–[6]. PCa progression is associated with altered expression of multiple oncogenes and tumor suppressors, and miRNAs may potentially regulate these genes; however, the relationship between miRNAs and PCa has only started to be elucidated in recent years [7]. More than 50 miRNAs are reported to be involved in PCa; however, most of the current data suggests that only a small number of these relate to the pathogenesis of PCa [8]. More miRNAs should be selected and studied in order to better understanding the progression of PCa.

To date, many articles investigated miRNA expression in PCa samples, however, the results were highly inconsistent [9]–[15]. No miRNA microarray detection results were reported from Chinese PCa samples so far. In the current study, we first screened the miRNAs related to PCa by miRNA microarrays, and according to the preliminary results, we found that microRNA-182 (miR-182) was overexpressed in PCa tissues. Studies showed that miR-182 could promote melanoma metastasis by repressing FOXO3 and microphthalmia-associated transcription factor [16], meanwhile, miR-183-96-182 cluster was overexpressed in prostate tissue and could regulate Zinc homeostasis in prostate cells [17]. MiR-182 was thought to be an important oncomiR. However, miR-182 could suppresse lung tumorigenesis through down regulation of RGS17 expression in vitro [18]. Furthermore, a new study showed that miR-182 and microRNA-200a could control G-protein subunit alpha-13 (GNA13) expression and cell invasion synergistically in PCa cells [19]. These inconsistent results indicate that the function of miR-182 is complex and further study is needed. In this study, we demonstrated that miR-182 promoted PCa PC-3 cells proliferation and invasion by directly targeting the 3′-UTR of N-myc downstream regulated gene 1 (NDRG1, NM_006096.3) mRNA. Our results suggest that miR-182 may play an important role in the development and progression of PCa.

## Materials and Methods

### Ethics Statement

The study was approved by the ethical board of the Second Hospital of Tianjin Medical University. All samples were obtained from patients who signed informed consent approving the use of their tissues for research purposes after operation.

### Prostate Tissue Samples

Five PCa tissues were collected after radical prostatectomy at the department of Urology of the hospital between January 2010 and December 2012. None of the patients had received neoadjuvant hormone therapy before the operation. Three benign prostatic hyperplasia (BPH) tissues were collected after suprapubic enucleation of the prostate. Fresh prostate tissues were sampled directly after surgical removal of the gland.These samples were immediately frozen in liquid nitrogen and used for miRNA microarray experiments. A diagnostic H&E section was prepared to verify tumor content and only cases with more than 90% tumor tissue were considered for further analysis.

### MiRNA Microarray Analysis

MiRNA isolation kits (Ambion) were used to isolate total RNA with enriched miRNAs from prostate tissue samples. Microarray assay was performed using a service provider (LC Sciences, Houston, TX, http://www.lcsciences.com) as described previously [20]. Those with fold change >2 or <−2 and P values <0.05 (t test) were considered as differentially expressed miRNAs. Real-time RT-PCR was used for the confirmation of some differentially expressed miRNAs.

### Cell Culture

Human PCa cell lines LNCap, PC-3, DU145, 22Rv1 and normal prostate epithelial cell line RWPE-1 were obtained from American Type Culture Collection (ATCC, Manassas, VA, USA) and kept in our laboratory. Cells were cultured in RPMI-1640 (Gibco) supplemented with 10% fetal-calf-serum and penicillin (100 U/ml). Cultures were maintained under an atmosphere containing 5% CO_2_.

### Total RNA Extraction and Real-time RT-PCR

Total RNA was extracted using Trizol Reagent (Invitrogen, CA, USA). cDNA was synthesized using M-MLV MicroRNA Reverse Transcription Kit (Promega, USA). Real-time RT-PCR was performed with SYBR® Premix Ex Taq™ (TaKaRa, Biotech Co., Ltd, Dalian, China). RT primer for miR-182 was 5′- GTCGTATCCAGTGCAGGGTCCGAGGTGCACTGGATACGACAGTGTGA -3′, PCR primer for miR-182 was 5′-TGCGGTTTGGCAATGGTAGAAC-3′(forward), 5′-CCAGTGCAGGGTCCGAGGT-3′ (reverse). The expression level were normalized to U6. RT primer for U6 was 5′- GTCGTATCCA GTGCAGGGTCCGAGG TGCACTGGATACGACAAAAT ATGG -3′, PCR primer for U6 was 5′- TGCGGGTGCTCGCTTCGGCAGC -3′(forward), 5′- CCAGTGCAGGGTCCGAGGT -3′ (reverse). PCR was performed under the following conditions: 94°C for 4 min, followed by 40 cycles at 94°C for 30 s, 50°C for 30 s and 72°C for 40 s. Each sample was run in triplicate.

### Western Blotting

Western blotting was performed to determine NDRG-1 protein expression. All proteins were resolved on an 8% SDS-denatured polyacrylamide gel and were then transferred onto a nitrocellulose membrane. Membranes were incubated with blocking buffer for 90 min at room temperature and then incubated with an antibody against NDRG-1 or Beta-tubulin with Blotto overnight at 4°C. The membranes were washed and incubated with a horseradish peroxidase (HRP)-conjugated secondary antibody. Protein expression was assessed by enhanced chemiluminescence and exposure to chemiluminescent film. The LabWorks image acquisition and analysis software (UVP, LLC) was used to quantify band intensities. All antibodies were purchased from Saier Biotechnology (Tianjin, China).

### Cell Transfection

Transfections were performed using Lipofectamine 2000 (Invitrogen, CA, USA) according to the manufacturer’s protocol. Briefly, PC-3 cells were plated in six well plates and grown without antibiotics to 60–80% confluence. Each well received 10 µl of lipofectamine reagent and 50 pmol of synthetic miR-182 mimics (GenePharma Co., Ltd, Shanghai, China), synthetic negative control miRNAs (miR-182 mimics-NC), synthetic miR-182-inhibitor sequence or synthetic miR-182-inhibitor negative control (miR-182 inhibitor-NC). Sequences of miR-182 mimics, miR-182 mimics-NC, miR-182-inhibitor and miR-182 inhibitor-NC were shown in table 1. All mimics and inhibitors were labelled with FAM (carboxyfluorescein). Six hours after transfection, PC-3 cells were observed using fluorescence microscope and serum free medium was changed to complete medium.

**Table 1:** Sequences of miR-182 mimics and inhibitors.

Fragments	Squences
miR-182 mimics (double strand)	5′-UUUGGCAAUGGUAGAACUCACACU-3′
	5′-UGUGAGUUCUACCAUUGCCAAAUU-3′
miR-182mimics-NC (double strand)	5′-UUCUCCGAACGUGUCACGUTT-3′
	5′-ACGUGACACGUUCGGAGAATT-3′
miR-182 inhibitor	5′-AGUGUGAGUUCUACCAUUGCCAAA-3′
miR-182 inhibitor-NC	5′-CAGUACUUUUGUGUAGUACAA-3′

### MTT Assay

PC-3 cells transfected with either miR-182 mimics or miR-182 mimics-NC, or miR-182-inhibitor and miR-182 inhibitor-NC were plated on 96-well plates at 1×10^4^ cells/well. Viable cells were measured 1, 2, 3, 4, and 5 days after plating. After incubation with 3-(4, 5- dimethylthiazolyl-2)-2, 5-diphenyltetrazolium bromide (MTT), the cells were lysed in 150 ml of 100% dimethylsulfoxide (DMSO) and UV-visible absorbance was read at 490 nm using the 96-well plate reader. Each sample was run in triplicate.

### Cell Cycle Analysis

PC-3 cells transfected with either miR-182 mimics or miR-182 mimics-NC, or miR-182-inhibitor and miR-182 inhibitor-NC were harvested 72 h after transfection, washed with cold phosphate buffered saline (PBS), and fixed in 1 ml of 70% ethanol. After overnight incubation at 4°C in ethanol, cells were washed in PBS and suspended in 500 ml propidine iodide (PI) 30 min before flow cytometry. Populations in G0–G1, S, and G2-M phase were measured by flow cytometry (BD Biosciences, USA) and the data were analyzed by using Multicycle-DNA Cell Cycle Analyzed Software.The measurement was performed in triplicate.

### Detection of Cell Early Apoptosis

PC-3 cells transfected with either miR-182 mimics or miR-182 mimics-NC, or miR-182-inhibitor and miR-182 inhibitor-NC were collected and fixed with 70% ethanol for the detection of early apoptosis. The dyeing of cells was performed according to the instructions of Annexin V-R-PE cell apoptosis detection kit (SouthernBiotech, USA). Flow cytometry (BD Biosciences, USA) was used to detect the percentage of early apoptosis. The measurement was performed in triplicate.

### Transwell Cell Invasion Assay

PC-3 cells transfected with either miR-182 mimics or miR-182 mimics-NC, or miR-182-inhibitor and miR-182 inhibitor-NC were collected. 5×10^4^ PC-3 cells were placed on the upper chamber of each insert coated with 50µl of 2 mg/ml Matrigel (growth factor reduced BD MatrigelTM matrix), and 600µl of RPMI 1640 with 20% FBS was added to the lower part of the chamber. After incubating for 24 hours, the chambers were disassembled, and the membranes were stained with a 2% crystal violet solution for 15 min and placed on a glass slide. Then, cells that had migrated across the membrane were counted in five random visual fields using a light microscope. All assays were performed three independent times in triplicate.

### miRNA Targets Prediction

The putative miRNA targets were predicted using the TargetScan (http://www.targetscan.org/vert_50/), miRBase (http://www.mirbase.org/index.shtml), and PicTar (http://pictar.mdc - berlin.de/) algorithms.

### Plasmid Construction

PmirGLO-Dual-luciferase reporter vector (7350 bp, Promega, Madison, WI, USA) was used to confirm the function of the putative miR-182 binding site in the NDRG1 3′-UTR. According to the potential miR-182 binding sequence of NDRG1 3′-UTR, a double - stranded sequence was obtained by annealing using two single-strands NDRG1-Top, (NheI) 5′-**CTAGC**TAGCGGCCGCTAGT CCTC AGAGAC ACCAAACTGCCAAAAG- 3′; NDRG1-Bot (SalI) 5′-**TCGAC**TTTTGGCA GTTTGGTGTC TCTGAGG ACTAGCGGCCGCTAG- 3′, and then cloned into the NheI/SalI sites of pmirGLO-Dual-luciferase reporter vector. Annealing was performed as: 95°C for 5 min and room temperature for 2 h. The reconstructed plasmid was confirmed by restriction endonuclease digestion and sequencing, and was named pmirGLO/NDRG1-UTR.

### Dual Luciferase Reporter Gene Assay

PC-3 cells were divided into five groups according to different transfection contents: a: (blank) pmirGLO/NDRG1-UTR; b: pmirGLO/NDRG1-UTR+miR-182 inhibitor-NC; c: pmirGLO/NDRG1-UTR+miR-182-inhibitor; d: pmirGLO/NDRG1-UTR+miR-182 mimics-NC; e: pmirGLO/NDRG1-UTR+miR-182 mimics. PC-3 cells were seeded in triplicate in 96-well plates, allowed to settle for 24 h and then co-transfected with different contents by using Lipofectamine 2000 (Invitrogen, CA, USA). Luciferase and Renilla activity were measured 48 h after transfection using the Dual LuciferaseReporter Assay Kit (Promega) according to the manufacturer’s instructions. Three independent experiments were performed and the data are presented as the mean ± SD. Luciferase activity values normalized to Renilla activity as relative light unit (RLU).

### Statistical Analysis

The two-tailed Student’s t-test was used to evaluate the significance of the differences between two groups; *P* values<0.05 were considered significant.

## Results

### MiR-182 was Upregulated in PCa Tissues

**Figure 1:**
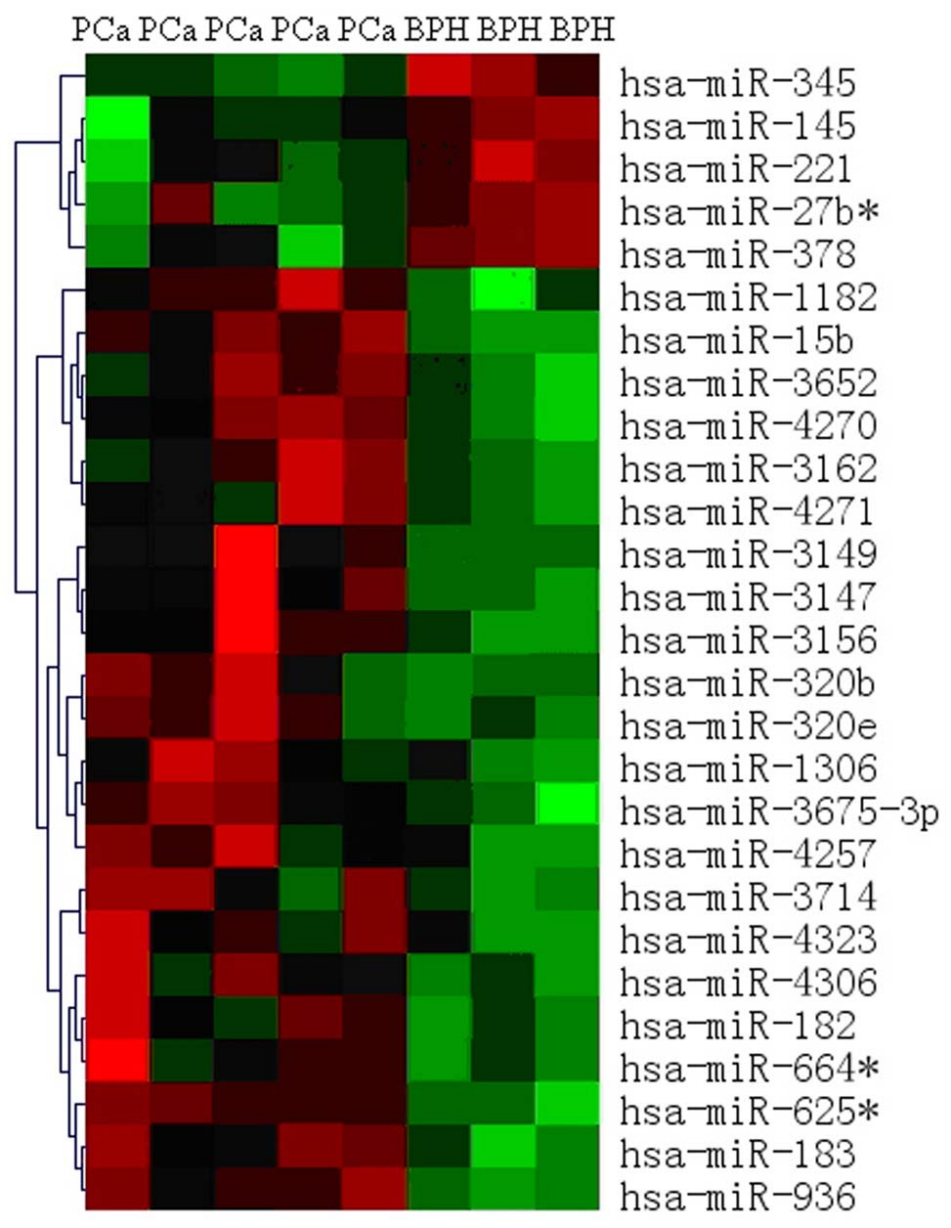
Significantly different expressed miRNAs between PCa and BPH by miRNA microarray analysis. In PCa tissues, five miRNAs were down-regulated and twenty-two miRNAs were up-regulated. miRNA-182 was up-regulated with fold change of 3.07 and further confirmed by real-time RT-PCR with fold change of 2.85.

After primary miRNA microarray analysis, in PCa tissues, five miRNAs (miR-345, miR-145, miR-221, miR-27b and miR-378) were down-regulated and twenty-two miRNAs were up-regulated (Figure 1). MiR-182 was up-regulated with fold change of 6.14 and further confirmed by real-time RT-PCR with fold change of 5.70. As the most significant differentially expressed miRNA between PCa and BPH tissues, miR-182 was chosen for further study. All the differentially expressed miRNAs were seen in Table S1.

### MiR-182 was Upregulated in PCa Cell Lines

**Figure 2:**
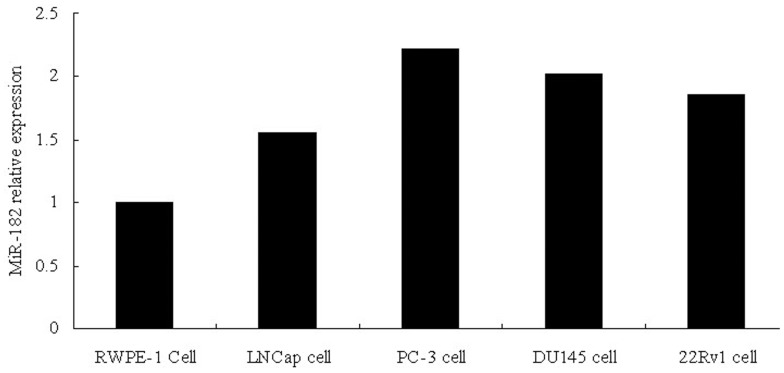
miR-182 expression in different prostate cells measured by real-time RT-PCR. Real-time RT-PCR show that the relative expression level of PC-3 cells is the highest in four prostate cancer cell lines and RWPE-1 has the lowest expression level. Values represent means from three separate experiments and error bars represent the SD.

Real-time RT-PCR analysis revealed that miR-182 expression was markedly increased in four common PCa cell lines tested (PC-3, DU145, 22Rv1 and LNCaP), compared to normal prostate epithelial RWPE-1 cell, indicating that miR-182 is upregulated in PCa cell lines and PC-3 has the highest expression level (Figure 2). Then PC-3 cells were chosen for further study.

### MiR-182 Expression Level is Significantly Changed after Transfection with Mimics or Inhibitors

**Figure 3:**
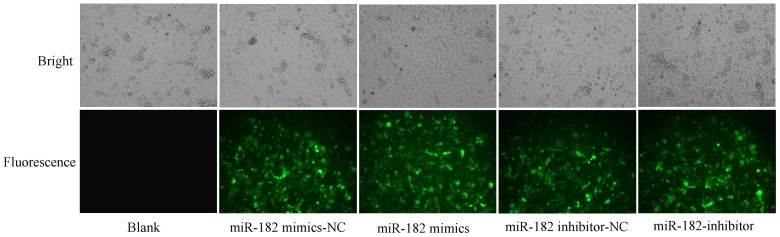
PC-3 cells were observed using fluorescence microscope after transfection with miR-182 mimics or inhibitors. Results show that the transfection rate is more than 80%. Representative images and randomly selected fields are shown.

**Figure 4:**
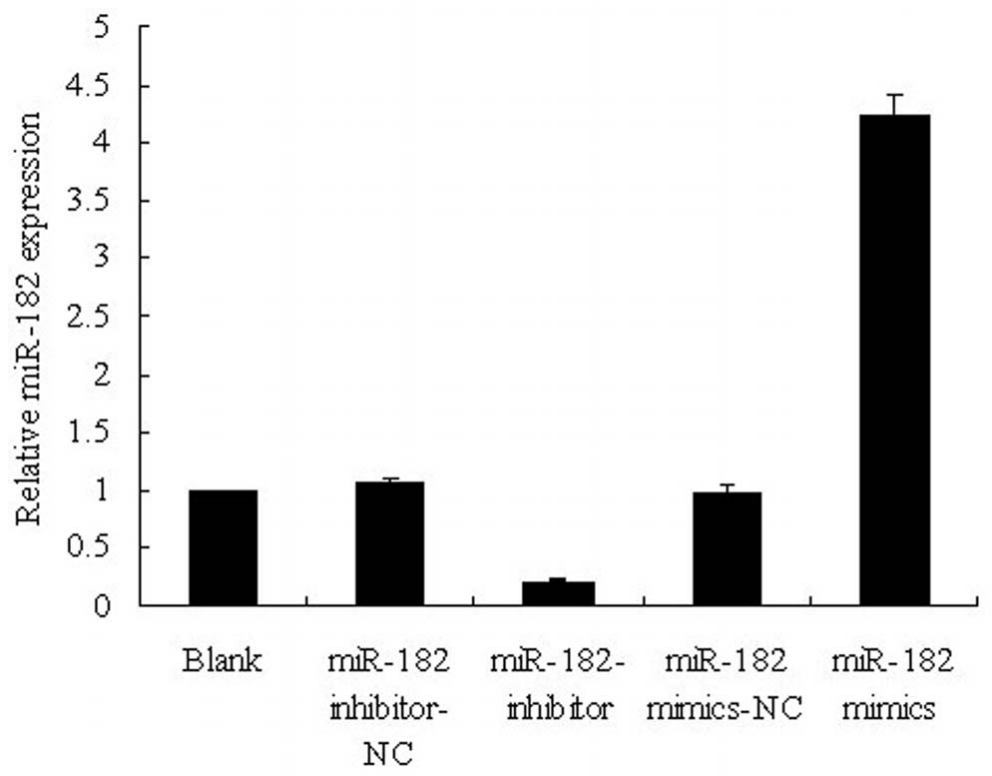
MiR-182 expression in PC-3 cells measured by real-time RT-PCR after transfection with miR-182 mimics or inhibitors. Blank presents blank control. The results show that the expression level is highest in miR-182 mimics group and lowest in miR-182 inhibitor group. Values represent means from three separate experiments and error bars represent the SD.

MiR-182 expression level was detected by real-time RT-PCR after transfection with mimics or inhibitors in PC-3 cells. Results showed that mimics or inhibitors were efficiently transfected (Figure 3) and the expression level is highest in miR-182 mimics group and lowest in miR-182 inhibitor group (Figure 4). These results indicated that these mimics or inhibitors can mimic or inhibit miR-182 effectively.

### Overexpression of miR-182 Promotes Proliferation in PC-3 Cells

**Figure 5:**
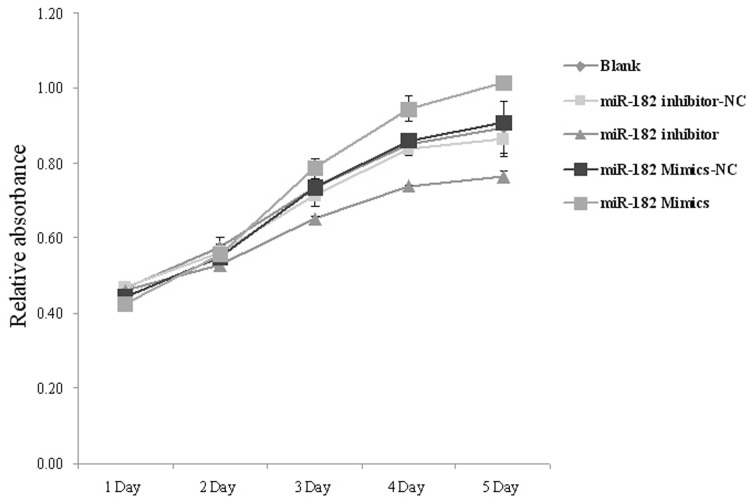
Viability of PC-3 cells transfected with miR-182 mimics or inhibitors was measured by MTT assays. Blank presents blank control. UV-visible absorbance was measured at 490 nm. The relative absorbance was significantly different 3 days after transfection (*P*<0.05). Values represent means from three separate experiments.

In order to investigate the function of miR-182 in PCa, we transfected miR-182 mimics or inhibitros into PC-3 PCa cells and measured cell proliferation. Using MTT assays, we observed that the growth rate of miR-182 overexpressing cells was dramatically increased, compared to miR-182 mimics -NC-transfected cells (*P*<0.05). On the contrary, after downregulation of miR-182 by an inhiobitor, the growth rate of PC-3 cells was dramatically decreased compared to miR-182 inhibitor -NC-transfected cells (*P*<0.05) (Figure 5). These results demonstrated that upregulation of miR-182 promotes the proliferation of PC-3 cells.

### Overexpression of miR-182 Promotes the G1/S Cell Cycle Transition in PC-3 Cells

**Figure 6:**
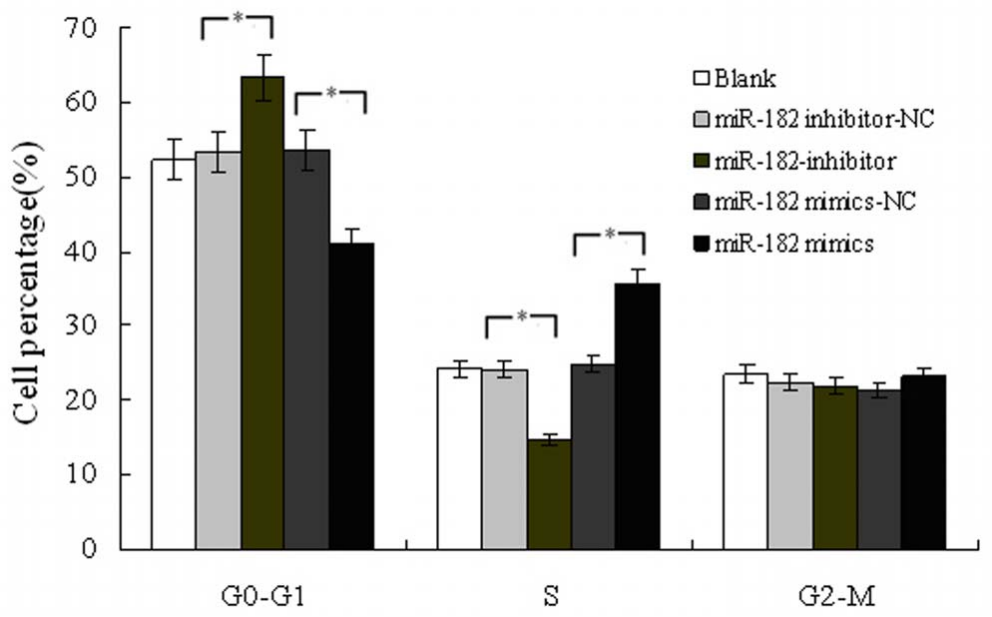
Analysis of cell cycle in PC3 cells after transfected with miR-182 mimics or inhibitors. Blank presents blank control. Values represent means from three separate experiments and error bars represent the SD. (*P<0.05).

We further investigated the effect of miR-182 on proliferation using flow cytometry. MiR-182-overexpressing PC-3 cells had a significantly lower percentage of cells in the G0/G1 phase and increased percentage of cells in the S phase, compared to miR-182 mimics-NC- transfected cells. On the contrary, after downregulation of miR-182 by an inhiobitor, PC-3 cells had a significantly higher percentage of cells in the G0/G1 phase and decreased percentage of cells in the S phase, compared to miR-182 inhibitor-NC-transfected cells (Figure 6). This data suggested that overexpression of miR-182 could promote the G1/S cell cycle transition, and may therefore, enhance the proliferation of PC-3 cells.

### Overexpression of miR-182 Reduces Early Apotosis of PC-3 Cells

**Figure 7:**
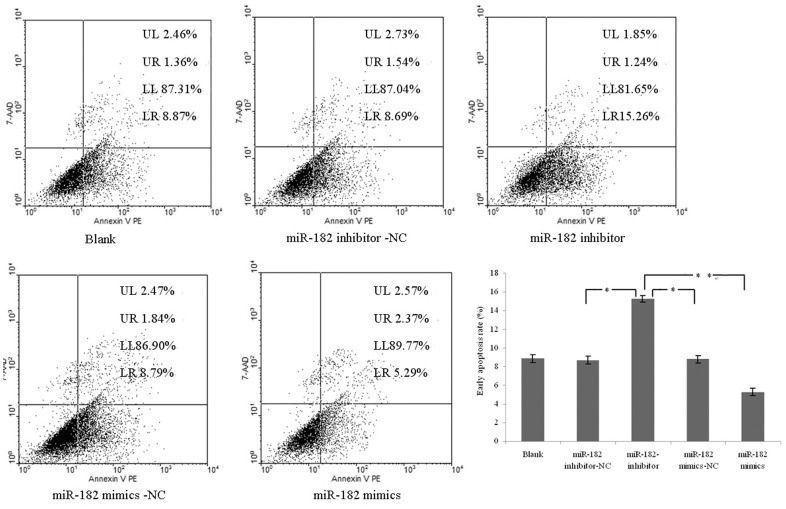
Early apoptosis detection of PC-3 cells transfected with miR-182 mimics or inhibitors. Blank presents blank control. The results show that the early apoptosis rate is lowest in miR-182 mimics group and highest in miR-182 inhibitor group (*P<0.05, **P<0.01). Representative images were shown and early apoptosis cells were indicated at Q4 (LR) area.

To investigate the possible regulative involvement of miR-182, early apoptosis of PC-3 cells were detected after transfection. Results showed that the early apoptosis rate of miR-182 overexpressing cells was dramatically decreased, compared to miR-182 mimics-NC-transfected cells. On the contrary, after downregulation of miR-182 by an inhiobitor, the early apoptosis rate of PC-3 cells was dramatically increased compared to miR-182 inhibitor-NC-transfected cells (Figure 7). These results demonstrated that upregulation of miR-182 reduces the early apoptosis of PC-3 cells.

### Overexpression of miR-182 Increases Invasion in PC-3 Cells

**Figure 8:**
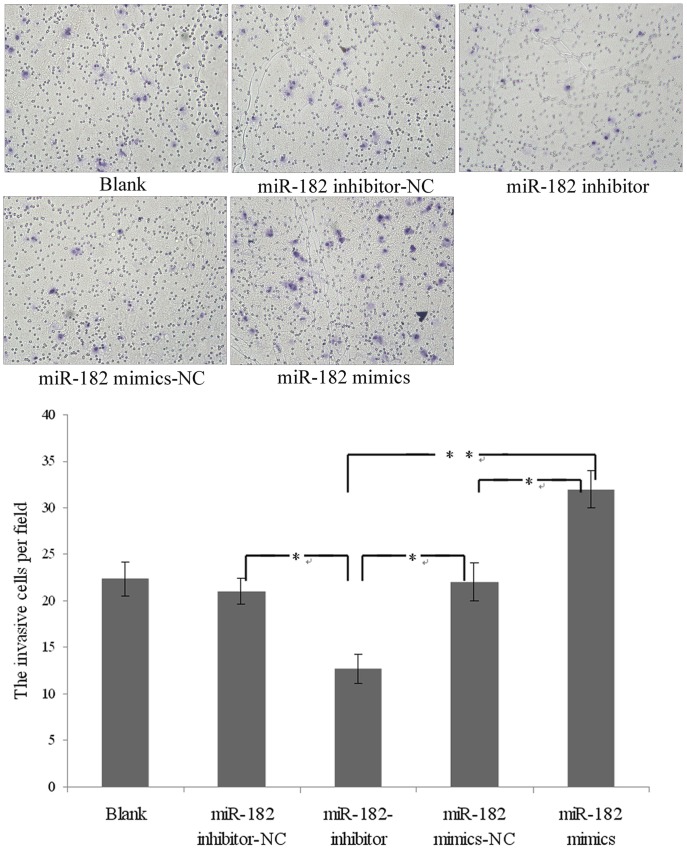
Transwell invasion assay. Invasion assays were performed with PC-3 cells transfected with miR-182 mimics or inhibitors. Blank presents blank control. Representative images and randomly selected fields are shown (*P<0.05, **P<0.01).

Overexpression of miR-182 significantly increased the invasive potential of PC-3 cells when transfected with miR-182 mimics compared with control cells in Transwell assay with Matrigel, and cells transfected with miR-182 inhibitor resulted in a significantly decresed invasive potential (Figure 8). These data suggest that miR-182 increases invasion in PC-3 cells *in vitro*.

### Identification of miR-182 Binding Sites in NDRG1 3′-UTR Region

**Figure 9:**
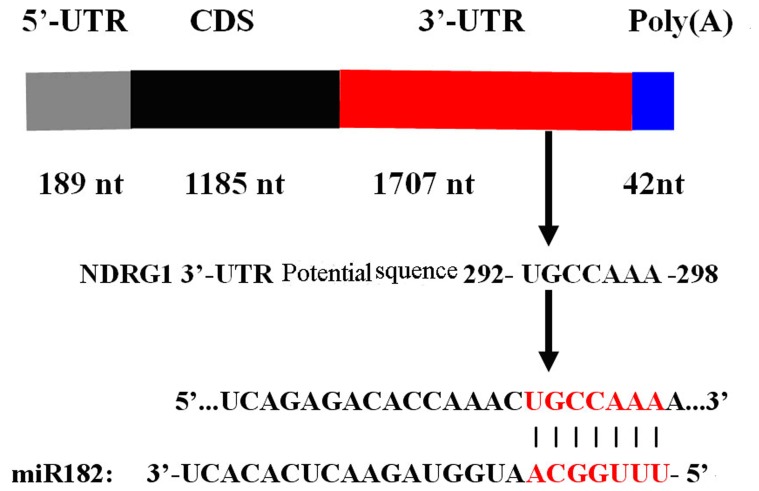
Schematic digram of NDRG1 3′-UTR region targeted by miR-182. NDRG1 3′-UTR region has only one highly conserved miR-182 binding sites after bioinformatic analysis.

After prediction with online miRNA Targets tools, many putative genes were predicted. Genes related with cell proliferation, apoptosis, invasion and metastasis were selected, including NDRG1. Furthermore, we found that NDRG1 was a metastasis supressor gene [21]–[25] or tumor suppressor gene [26], and NDRG1 was necessary for p53-dependent apoptosis [27]. Our study also showed that NDRG1 was downregulated in PCa tissues and PC-3 cells compared with BPH tissues and RWPE-1 cells (data not shown). Finally, NDRG1 was selected for further study. Next, we found that NDRG1 3′-UTR region has only one highly conserved miR-182 binding sites (Figure 9).

### MiR-182 Directly Targets the Metastasis Supressor Gene NDRG1 in PC-3 Cells

**Figure 10:**
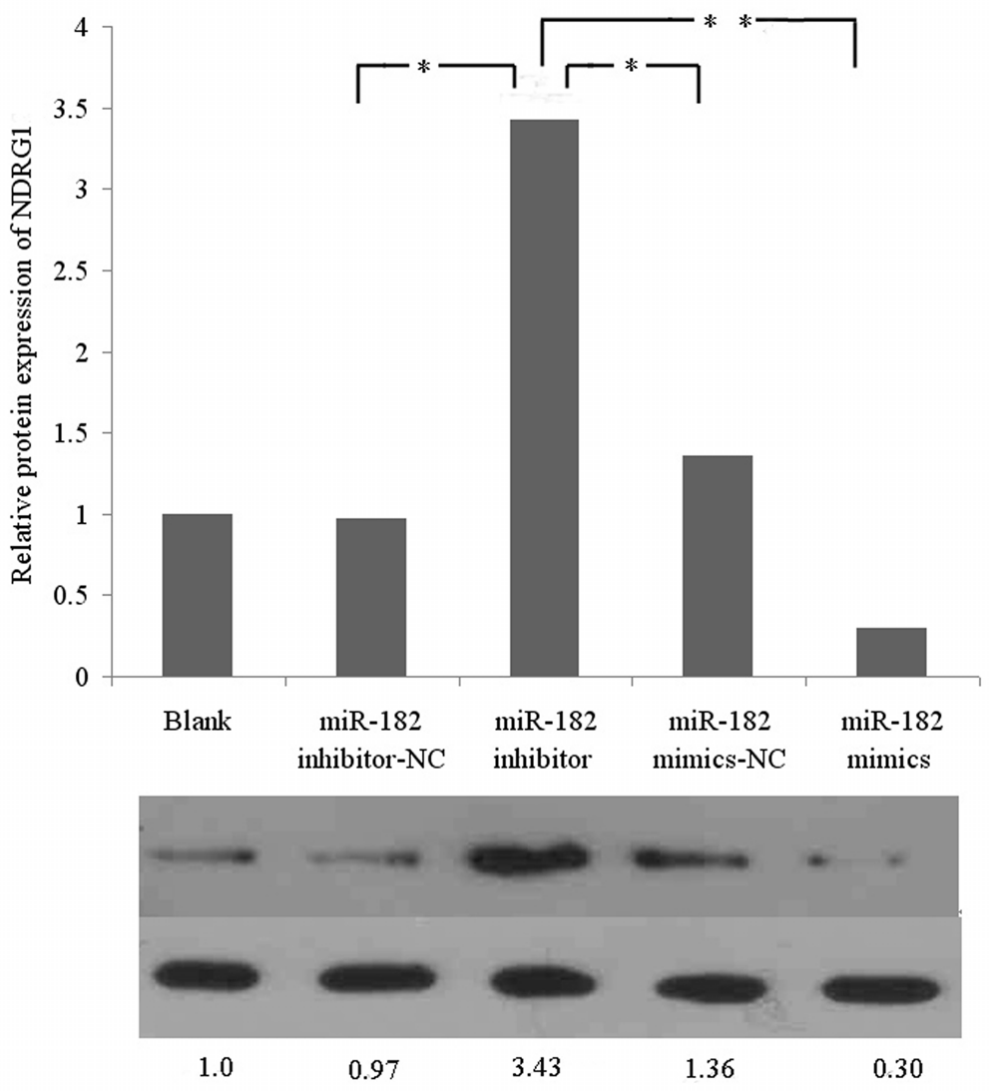
Western Blotting results of NDRG1 protein expression in PC-3 cells after transfection with miR-182 mimics or inhibitors. Blank presents blank control. Overexpression of miR-182 significantly decreased the protein expression levels of NDRG1 and downregulation of miR-182 dramatically increased the protein expression levels of NDRG1 (*P<0.05, **P<0.01). Values represent means from three separate experiments and error bars represent the SD.

Western Blotting results showed that overexpression of miR-182 in PC-3 cells significantly decreased the protein expression levels of NDRG1 after transfection with miR-182 mimics, compared to miR-182 mimics -NC-transfected cells. On the contrary, after downregulation of miR-182 by an inhiobitor, the protein expression levels of NDRG1was dramatically increased compared to miR-182 inhibitor-NC-transfected cells (Figure 10), indicating that NDRG1 is a potential miR-182 target gene.

**Figure 11:**
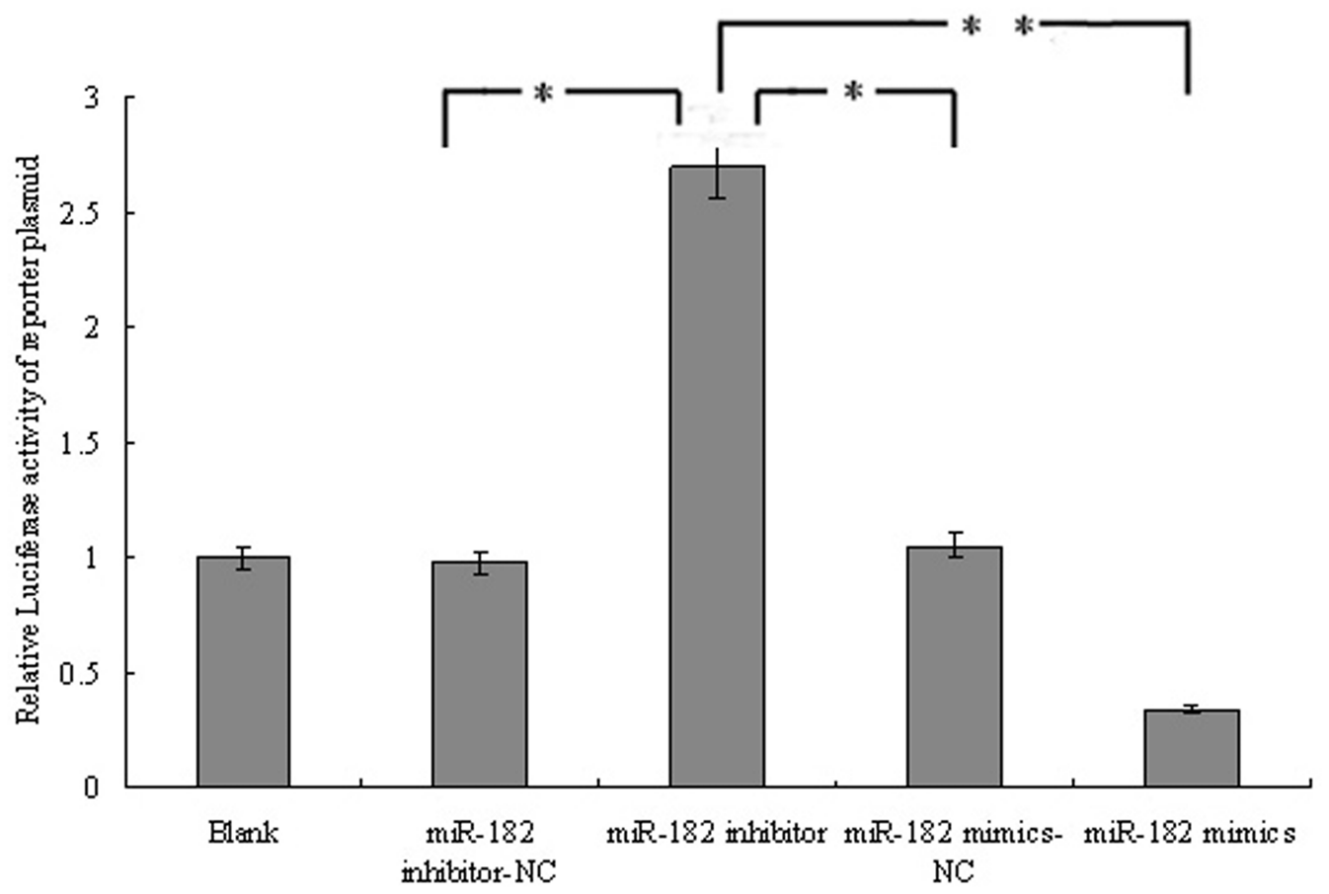
Relative luciferase activity of the pmirGLO/NDRG1-UTR in PC-3 cells. Luciferase activity of the pmirGLO/NDRG1-UTR was dramatically inhibited in miR-182 mimics group and significantly increased in miR-182 inhibitors group, compared to the control groups (*P<0.05, **P<0.01).

To confirm the function of the putative miR-182 binding site in the NDRG1 3′-UTR, we synthesized the double - stranded sequence which included the miR-182 binding site and cloned into the luciferase reporter plasmid. Luciferase activity of the pmirGLO/NDRG1-UTR was dramatically inhibited by overexpression of miR-182 with cotransfection with miR-182 mimics and significantly increased by downregulation of miR-182 with cotransfection with miR-182 inhibitors in PC-3 cells, compared to the control plasmids (Figure 11), suggesting that miR-182 specifically targets the NDRG1 3′-UTR.

## Discussion

Over the past few years, hundreds of miRNAs have been shown to play important roles in regulating gene expression through degradation of mRNA or repression of translation in a variety of model systems [28]–[29]. Evidence suggests that miRNAs may function as a novel class of both tumorigenic and tumor-suppressing genes [30]. In this study, we first prepared ten PCa samples and ten BPH samples, however, only five PCa samples and three BPH samples met the screening standard of microarray test. After miRNA microarray analysis and real-time RT-PCR confirmation, we demonstrated that miR-182 is upregulated in Chinese PCa tissues, compared to BPH tissues. The upregulation result of miR-182 was consistent with the study by Schaefer A et al [14]. Then we revealed that miR-182 expression was markedly increased in four common PCa cell lines tested (PC-3, DU145, 22Rv1 and LNCaP), compared to normal prostate epithelial RWPE-1 cell, indicating that miR-182 is upregulated in PCa cell lines and PC-3 has the highest expression level. Then PC-3 cells were chosen for further study. Next we prove that miR-182 specifically targets the NDRG1 3′-UTR in PC-3 cells.

Previous studies revealed that NDRG1 was a metastasis supressor gene or tumor suppressor gene of PCa [21], [24], [25]. Our study also showed that NDRG1 was downregulated in PCa tissues and PC-3 cells compared with BPH tissues and RWPE-1 cells (data not shown). Further functional experiment showed that downregulation of NDRG1 could increase the proliferation and invasion of PC-3 cells, and upregulation of NDRG1 could decrease the proliferation and invasion of PC-3 cells (data not shown). Studies showed that the molecular regulation of NDRG1 invovles PTEN [21], [26], P21 [22], Wnt-β-catenin [23], activating transcription factor 3 [24], ATF3-NF kappaB complex [25] and p53 [27]. However, there was no report about NDRG1 regulated by microRNA in PCa to promote proliferation clearly and directly yet. It is the first for us to uncover the new relationship of regulation of NDRG1 and microRNA in PCa. We observed that miR-182 was significantly overexpressed in PCa cell lines, compared to RWPE-1. Furthermore, ectopic overexpression of miR-182 significantly promotes the proliferation, increases the invasion, promotes the G1/S cell cycle transition and reduces early apotosis of PC-3 cells, while suppression of miR-182 decreased the proliferation and invasion, inhibits the G1/S cell cycle transition and increase early apotosis of PC-3 cells. In order to explore the mechanism of miR-182, we identified NDRG1 as a putative miR-182 target gene using bioinformatic analysis, and confirmed that NDRG1 is a direct target of miR-182 by dual luciferase reporter gene assay. These results showed that miR-182 increases the proliferation and invasion of PCa PC-3 cells by directly targeting the NDRG1 3′-UTR to downregulate NDRG1.

In recent years, NDRG1 has been described as a potential tumor suppressor gene in various human cancers, and may be associated with tumor aggressiveness and metastasis [31]–[34]. However, the silencing machanism of NDRG1 is still not clear. One way in which cancer cells silence tumor suppressor gene expression are epigenetic alterations, which include DNA hypermethylation of promoter CpG islands and/or changes in associated post-translational histone modifications leading to transcriptional repression [35]. The promoter of NDRG1 contains a large CpG island, raising the possibility that it could be silenced by DNA hypermethylation in cancer. Therefore, researchers begin to study the epigenetic regulation of NDRG1 recently. Angst et al [36] study showed that NDRG1 expression can be regulated by the pharmacologic inhibition of DNA methylation and histone deacetylation in pancreatic cancer cells. However, no methylation of the CpG island of the NDRG1 promoter was found in pancreatic cancer cells, suggesting an indirect mechanism for NDRG1 de-repression by these treatments. Li and Chen [37] study showed that the silencing of NDRG1 in colon cancer cell lines SW620 and SW480 was due to histone modifications, other than promoter hypermethylation. However, Chang et al [38] study showed that the decreased expression of NDRG1 mRNA and protein in gastric cancer cell lines and tissues was due to methylation of NDRG1 gene promoter. These studies suggest that the epigenetic regulation of NDRG1 is different in different types of cancer cells. In this study, we showed that NDRG1 could be directly targeted by miR-182 and uncovered a new epigenetic regulation of NDRG1, suggesting that upregulation of miR-182 may provide an alternative mechanism for the reduced expression of the NDRG1 tumor suppressor protein in PCa cells.

Further research is still required to examine whether other miRNAs or signaling pathways can regulate NDRG1 in PCa, because we could not exclude that there might be other microRNAs, not found yet, to play an important role in regulating NDRG1 in PCa, and whether miR-182 can target other members of the NDRG1 family. For example, it would be of interest to know whether other pathways are involved in the antiproliferative effect of NDRG1 and investigate what other components of the malignant phenotype are determined by NDRG1 and miRNAs in PCa cells, and these issues are currently under further investigation in our laboratory.

### Conclusions

In summary, the key finding of the current study is that miR-182 can increase the proliferation of PCa cell lines by targeting NDRG1. This data indicates that miR-182 plays an essential role in the regulation of PCa cell proliferation and may function as an onco-miRNA. MiR-182 may sever as a novel therapeutic target for the treatment of PCa.

## Supporting Information

Table S1 **Differentially expressed miRNAs between PCa and BPH tissues.** Bold miRs were downregulated miRs in PCa tissues**.**
(DOC)
