# Supplementary material for: Overexpressed MicroRNA-182 Promotes Proliferation and Invasion in Prostate Cancer PC-3 Cells by Down-Regulating N-myc Downstream Regulated Gene 1 (NDRG1)
Source: PLoS One. 2013 Jul 16;8(7):e68982. doi: 10.1371/journal.pone.0068982 (PMC3712934; doi:10.1371/journal.pone.0068982)
Supplement: Table S1 — Differentially expressed miRNAs between PCa and BPH tissues. Bold miRs were downregulated miRs in PCa tissues. (DOC) [file pone.0068982.s001.doc]

Table S1. Differentially expressed miRNAs between PCa and BPH tissues. Bold miRs were downregulated miRs in PCa tissues.

| miRs | P value | Fold change |
| --- | --- | --- |
| hsa-miR-182 | 1.98E-02 | 6.14 |
| **hsa-miR-221** | 2.60E-02 | -5.82 |
| hsa-miR-320e | 2.65E-02 | 2.88 |
| hsa-miR-320b | 2.71E-02 | 2.66 |
| **hsa-miR-145** | 2.97E-02 | -5.78 |
| **hsa-miR-27b*** | 1.13E-02 | -2.24 |
| hsa-miR-183 | 1.21E-02 | 5.08 |
| **hsa-miR-378** | 1.29E-02 | -2.36 |
| hsa-miR-4306 | 1.57E-02 | 2.60 |
| hsa-miR-3162 | 1.73E-02 | 5.30 |
| hsa-miR-3147 | 2.44E-02 | 2.28 |
| hsa-miR-3156 | 2.55E-02 | 2.84 |
| hsa-miR-664* | 2.86E-02 | 2.88 |
| hsa-miR-4271 | 3.44E-02 | 4.14 |
| hsa-miR-625* | 3.57E-02 | 2.78 |
| hsa-miR-3652 | 3.66E-02 | 2.98 |
| hsa-miR-1306 | 3.76E-02 | 3.54 |
| hsa-miR-3714 | 3.81E-02 | 2.04 |
| hsa-miR-4323 | 3.85E-02 | 2.12 |
| **hsa-miR-345** | 3.98E-02 | -2.6 |
| hsa-miR-1182 | 4.16E-02 | 3.66 |
| hsa-miR-4257 | 4.24E-02 | 2.16 |
| hsa-miR-3675-3p | 4.74E-02 | 2.98 |
| hsa-miR-3149 | 4.85E-02 | 3.14 |
| hsa-miR-15b | 2.28E-03 | 3.42 |
| hsa-miR-936 | 2.30E-03 | 4.14 |
| hsa-miR-4270 | 8.24E-03 | 5.14 |
